# Supplementary material for: Association of multidrug-resistant bacteria and clinical outcomes in patients with infected diabetic foot in a Peruvian hospital: A retrospective cohort analysis
Source: PLoS One. 2024 Jun 4;19(6):e0299416. doi: 10.1371/journal.pone.0299416 (PMC11149844; doi:10.1371/journal.pone.0299416)
Supplement: S1 Fig — (DOCX) [file pone.0299416.s001.docx]

**S1 Fig. Diagram of distribution of outcomes in the study's patients.**

^to^Primary outcome: Death and/or major amputation.
