# Supplementary material for: Association of multidrug-resistant bacteria and clinical outcomes in patients with infected diabetic foot in a Peruvian hospital: A retrospective cohort analysis
Source: PLoS One. 2024 Jun 4;19(6):e0299416. doi: 10.1371/journal.pone.0299416 (PMC11149844; doi:10.1371/journal.pone.0299416)
Supplement: S1 Table — (DOCX) [file pone.0299416.s002.docx]

**S1 Table. Categories and agents used to define *Staphylococcus aureus* MDR, XDR, and PDR.**

| Antimicrobial category | Antimicrobial agent | Acquired resistance  Yes No |
| --- | --- | --- |
| Aminoglycosides | Gentamicin |  |
| Ansamycins | Rifampicin |  |
| Anti MRSA cephalosporins | Ceftaroline |  |
| Antistaphylococcal beta-lactams or (cephamycin) | Oxacillin (or cefoxitin) |  |
| Fluoroquinolones | Ciprofloxacin  Moxifloxacin |  |
| Folate inhibitors | Cotrimoxazole |  |
| Fucidin | Fusidic acid |  |
| Glycopeptides | Vancomycin  Teicoplanin  Telavancin |  |
| glycylcyclines | Tigacycline |  |
| Lincosamides | Clindamycin |  |
| Lipopeptides | Daptomycin |  |
| Macrolides | Erythromycin |  |
| Oxazolidinones | Linezolid |  |
| Phenicoles | Chloramphenicol |  |
| Phosphonic Acid | Fosfomycin |  |
| Streptogramins | Quinupristin  Dalfopristin |  |
| Tetracycline | Tetracycline  Doxycycline  Minocycline |  |

MDR (1 or more of the following): 1. MRSA 2. Resistant to ≥ 1 agent in ≥ 3 categories.

XDR : Resistant to ≥ 1 agent in almost all but ≤ 2 categories.

PDR: Resistant to everything.

Oxacillin or Cefoxitin, represents all the other beta-lactams.
