# Supplementary material for: Association of multidrug-resistant bacteria and clinical outcomes in patients with infected diabetic foot in a Peruvian hospital: A retrospective cohort analysis
Source: PLoS One. 2024 Jun 4;19(6):e0299416. doi: 10.1371/journal.pone.0299416 (PMC11149844; doi:10.1371/journal.pone.0299416)
Supplement: S2 Table — (DOCX) [file pone.0299416.s003.docx]

**S2 Table. Categories and agents used to define *Enterococcus sp* MDR, XDR and PDR.**

| Antimicrobial category | Antimicrobial agent | Natural resistance | Acquired resistance  Yes No |
| --- | --- | --- | --- |
| Aminoglycosides | Gentamicin |  |  |
| Streptomycins | Streptomycin |  |  |
| Carbapenems | Imipinemem  Meropenem  Doripenem | *E.faecium* |  |
| Fluoroquinolones | Ciprofloxacin  Levofloxacin  Moxifloxacin |  |  |
| Glycopeptides | Vancomycin  Teicoplanin |  |  |
| Glycylcyclines | Tigacycline |  |  |
| Lipopeptides | Daptomycin |  |  |
| Oxazolidinones | Linezolid |  |  |
| Penicillins | Ampicillin |  |  |
| Streptogramins | Quinupristin  Dalfopristin | *E. faecalis* |  |
| Tetracycline | Tetracycline  Doxycycline  Minocycline |  |  |

MDR: Resistant to ≥ 1 agent in ≥ 3 categories.

XDR : Resistant to ≥ 1 agent in almost all but ≤ 2 categories.

PDR: Resistant to everything.

Natural resistances are not taken into account for the definitions.
