# Supplementary material for: Association of multidrug-resistant bacteria and clinical outcomes in patients with infected diabetic foot in a Peruvian hospital: A retrospective cohort analysis
Source: PLoS One. 2024 Jun 4;19(6):e0299416. doi: 10.1371/journal.pone.0299416 (PMC11149844; doi:10.1371/journal.pone.0299416)
Supplement: S3 Table — (DOCX) [file pone.0299416.s004.docx]

**S3 Table**. **Categories and agents used to define *Enterobacteriaceae* MDR, XDR and PDR.**

| Antimicrobial category | Antimicrobial agent | Natural resistance | Acquired resistance  Yes No |
| --- | --- | --- | --- |
| Aminoglycosides | Gentamicin  Tobramycin  Amikacin  Netilmicin | *Providencia rettgeri, Providencia stuarti*  *P. rettgeri, P. stuarti*  *P. rettgeri, P. stuarti* |  |
| Anti MRSA cephalosporins | Ceftaroline (approved only for *E. coli, K. pneumoniae and K. oxytoca*) |  |  |
| Antipseudomonal penicillins + beta-lactamase inhibitors | Ticarcillin/ Ac. clavulamic  Piperacillin/Tazobactam | *Escherichia hermanii*  *E. hernanii* |  |
| Carbapenems | Ertapenem  Imipenem  Meropenem  Doripenem |  |  |
| Non-extended spectrum cephalosporins: 1st and 2nd generation | Cefazolin | *Citrobacter freundii, Enterobacter aerogenes, Enterobacter cloacae,*  *Hafnia alvei, Morganella morganii,*  *Proteus penneri, Proteus vulgaris,*  *P. rettgeri, P. stuartii, Serratia marcescens.* |  |
|  | Cefuroxime | *C. freundii, E. aerogenes, P. vulgaris,*  *S. marcescens* |  |
| Extended spectrum cephalosporins: 3rd and 4th generation | Cefotaxime or ceftriaxone  Ceftazidime  Cefepime |  |  |
| Cephamycins | Cefoxitin  Cefotetan | *C. freundii, E. aerogenes, E. cloacae, H. alvei* |  |
| Fluoroquinolones | Ciprofloxacin |  |  |
| Folate inhibitors | Cotrimoxazole |  |  |
| Fucidin | Fusidic acid |  |  |
| Glycylcyclines | Tigacycline | *M.morganii, Proteus mirabilis,*  *P. penneri, P. vulgaris, P. rettgeri,*  *P. stuarti* |  |
| Monobactams | Aztreonam |  |  |
| Penicillins | Ampicillin | *Citrobacter kaseri, C. freundii,*  *E. aerogenes, E cloacae, H. alvei,*  *Klebsiella spp, M. morganii, P. penneri,*  *P. vulgaris. P. rettgeri, P. stuarti,*  *S. marcescens* |  |
| Penicillins + beta lactamase inhibitors | Amoxicillin-Clavulamic acid | *C. freundii, E. aerogenes, E cloacae,*  *H. alvei, M. morganii, P. rettgeri,*  *P. stuarti, S. marcescens* |  |
|  | Ampicillin sulbactam | *C. freundii, C. kaseri, E. aerogenes,*  *E cloacae, H. alvei, P. rettgeri,*  *S. marcescens* |  |
| Phenicoles | Chloramphenicol |  |  |
| Phosphonic Acid | Fosfomycin |  |  |
| Polymyxins | Colistin | *M. morganii, P. mirabilis, P. penneri,*  *P. vulgaris. P. rettgeri, P. stuarti,*  *S. marcescens* |  |
| Tetracycline | Tetracycline | *M. morganii, P. mirabilis, P. penneri,*  *P. vulgaris. P. rettgeri, P. stuarti* |  |
|  | Doxycycline  Minocycline | *M. morganii, P. penneri, P. vulgaris.*  *P. rettgeri, P. stuarti* |  |

MDR: Resistant to ≥ 1 agent in ≥ 3 categories.

XDR : Resistant to ≥ 1 agent in almost all but ≤ 2 categories.

PDR : Resistant to everything.

Natural resistances are not taken into account for the definitions.
