# Supplementary material for: Association of multidrug-resistant bacteria and clinical outcomes in patients with infected diabetic foot in a Peruvian hospital: A retrospective cohort analysis
Source: PLoS One. 2024 Jun 4;19(6):e0299416. doi: 10.1371/journal.pone.0299416 (PMC11149844; doi:10.1371/journal.pone.0299416)
Supplement: S4 Table — (DOCX) [file pone.0299416.s005.docx]

**S4 Table. Categories and agents used to define *Pseudomonas aeuruginosa* MDR, XDR and PDR.**

| Antimicrobial category | Antimicrobial agent | Acquired resistance  Yes No |
| --- | --- | --- |
| Aminoglycosides | Gentamicin  Tobramycin  Amikacin  Netilmicin |  |
| Antipseudomonal Carbapenems | Imipenem  Meropenem  Doripenem |  |
| Antipseudomonal Cephalosporins | Ceftazidime  Cefepime |  |
| Antipseudomonal Fluoroquinolones | Ciprofloxacin  Levofloxacin |  |
| Antipseudomonal penicillins + beta-lactamase inhibitors | Ticarcillin/ clavulamic acid  Piperacillin/Tazobactam |  |
| Folate inhibitors | Cotrimoxazole |  |
| Monobactams | Aztreonam |  |
| Phosphonic Acid | Fosfomycin |  |
| Polymyxins | Colistin  Polymyxin B |  |

MDR: Resistant to ≥ 1 agent in ≥ 3 categories.

XDR : Resistant to ≥ 1 agent in almost all but ≤ 2 categories.

PDR : Resistant to everything.
