# Supplementary material for: Association of multidrug-resistant bacteria and clinical outcomes in patients with infected diabetic foot in a Peruvian hospital: A retrospective cohort analysis
Source: PLoS One. 2024 Jun 4;19(6):e0299416. doi: 10.1371/journal.pone.0299416 (PMC11149844; doi:10.1371/journal.pone.0299416)
Supplement: S6 Table — (DOCX) [file pone.0299416.s007.docx]

Table S6. CPT codes that include the major leg amputation procedure:

| *Major amputation* | *CPT* Code | Description of Procedure |
| --- | --- | --- |
| Below knee amputation | 27880 | Amputation leg through tibia and fibula |
|  | 27881 | Amputation leg through the tibia and fibula with immediate fitting technique including application of first cast |
|  | 27882 | Amputation leg through the tibia and fibula, open circulatory (guillotine) |
|  | 27886 | Amputation leg through the tibia and fibula, re-amputation |
| Above knee amputation | 27295 | Disarticulation of hip |
|  | 27590 | Amputation, thigh, through femur, any level |
|  | 27591 | Amputation, thigh, through the femur, any level, an immediate fitting technique including the first cast |
|  | 27592 | Amputation, thigh, through femur, any level, open, circular (guillotine) |
|  | 27596 | Amputation, thigh, through femur, any level, re-amputation |
|  | 27598 | Disarticulation at the knee |
