# Supplementary material for: Association of multidrug-resistant bacteria and clinical outcomes in patients with infected diabetic foot in a Peruvian hospital: A retrospective cohort analysis
Source: PLoS One. 2024 Jun 4;19(6):e0299416. doi: 10.1371/journal.pone.0299416 (PMC11149844; doi:10.1371/journal.pone.0299416)
Supplement: S7 Table — (DOCX) [file pone.0299416.s008.docx]

**S7 Table. Scales to assess the characteristics of the DFI.**

|  | MEGGIT WAGNER* | | UNIVERSITY OF TEXAS | |  | PEDIS | SINBAD | | SAINT ELIAN* | |
| --- | --- | --- | --- | --- | --- | --- | --- | --- | --- | --- |
| Depth | 0  1  2  3 | Pre-ulcer  TCSC Skin  Tendon, capsule  Osteitis | 0  1  2  3 | No  Skin, TCSC  Tendon, capsule  Arctic, bone | 1  2  3 | Skin, TCSC  Tendon, capsule  Joint, bone | 0  1 | TCSC Skin  Rest | 1  2  3 | Skin  Tendon, fascia, muscle  Joint, bone |
| Ischemia | 4  5 | Partial necrosis  Total necrosis | 0  1 | No  Absence of pulse & ABI<0.8 | 1  2  3 | Normal^a^  Claudication^a^  Critical ischemia^a^ | 0  1 | Palpable pulse  No pulse | 0  1  2  3 | No^b^  Mild^b^  Moderate^b^  Severe^b^ |
| Infection |  |  | 0  1 | No infection  Pus or more than 2 signs of infection | 1  2  3  4 | IDSA^c^  No  Mild  Moderate  Severe | 0  1 | No infection  Any signs of infection | 0  1  2  3 | IDSA^c^  No  Mild  Moderate  Severe |
| Neuropathy |  |  |  |  | 1  2 | Normal  PSP^d^ | 0  1 | Normal  PSP^d^ | 0  1  2  3 | **No**  PSP^d^  Anesthesia  Charcot |
| Extension |  |  |  |  |  | Area  ___ x ____ | 0  1 | <1  >1 | 1  2  3 | <10  11-40  >40 |
| Starting place |  |  |  |  |  |  | 0  1 | Forefoot  Half-retropie | 1  2  3 | Phalanx  Metatarsus  Tarsus |
| N° of zones |  |  |  |  |  |  |  |  | 1  2  3 | A zone  Two zones  Whole foot |
| Location |  |  |  |  |  |  |  |  | 1  2  3 | Dorsoplantar  Lateromedial  More than 2 |
| Edema |  |  |  |  |  |  |  |  | 0  1  2  3 | No  Periwound  Unilateral  Bilateral |
| Wound phase |  |  |  |  |  |  |  |  | 1  2  3 | Epithelialized  Granulated  Inflamed |

For informational purposes, we describe the components and categories of the scales used and others that were not available. * Used in the research.

^a^ISCHEMIA PEDIS: Without symptoms or signs of peripheral arterial disease ABI 0.9 - 1.1 (1). With symptoms or signs of peripheral arterial disease, but without ischemia. Intermittent cludication, ABI <0.9, ankle pressure >50 mm Hg (2). Critical limb ischemia. Ankle pressure <50 mm Hg (3).

^b^SAINT ELIAN ISCHEMIA: Without symptoms, normal pulse & ABI 0.9-1.2 (0) **Mild:** With or without symptoms &↓ pulses & ABI 0.7-0.89 (1) **Moderate:** ↓↓ pulses & ABI 0.5-0.69 (2) **Severe:** necrosis ischemic & no pulses & ABI <0.5 & studies showing ischemia (3)

^c^IDSA: No (0). **Mild:** 0.5- 2cm erythema, edema, heat, pain, and purulent discharge (1). **Moderate:** erythema>2cm, abscess, necrosis, fasciitis, and osteomielitis (2). **Severe:** Systemic inflammatory response, hyper-hypoglycemia (3).

^d^PSP: Insensitivity to 10g monofilament in 2 of 3 places or alteration of vibration sensitivity by means of 128 Hz tuning fork in 1st MTT head.
