# Supplementary material for: Association of multidrug-resistant bacteria and clinical outcomes in patients with infected diabetic foot in a Peruvian hospital: A retrospective cohort analysis
Source: PLoS One. 2024 Jun 4;19(6):e0299416. doi: 10.1371/journal.pone.0299416 (PMC11149844; doi:10.1371/journal.pone.0299416)
Supplement: S8 Table — (DOCX) [file pone.0299416.s009.docx]

**S8 Table. Data collection instrument.**

1. **Clinical history**

| CV risk factors | a) HBP (0) No (1) Yes b) Dyslipidemia (0) No (1) Yes  c) Tobacco consumption (0) No (1) Yes d) BMI (kg/m^2^) : __________ |
| --- | --- |
| Sick time | a) Time of DM2 (years) :____ b) Time of ulcer (weeks) : ______ |
| Ulcer | a) New (0) b) Recurrence (lesion in the same area without epithelization)  c) Relapse (lesion in the same epithelized area) |
| Previous infection in the same wound | a) No (0) b) Ambulatory N° of reinfection:____ Time to cure:_______  c) Hospitalized N° Hospitalizations:_____ Hospitalized time:_______ |
| Previous ATB use | a) No (0) b) Cipro (1) c) Ceftri (2) d) Imipenem (3) e) Other:___________ |
| Previous amputation | Minor a.No b.Yes Major IC a. No b.Yes Major SC a. No b. Yes |
| Microcomplications | Diabetic nephropathy. a. No b. Yes  Creatinine clearance <60 a. No b. Yes  Diabetic retinopathy or laser treatment a. No b. Yes |
| Macrocomplications | Cerebrovascular disease a. No b. Yes Coronary heart disease a. No b. Yes |
| Laboratory | HbA1c :_______ Albumin: _______ Hb:_______ LDL c :_______  HDL c: _______ Creatinine:_______ Glucose:______ Leukocytes: _______  Lymphocytes:______ CRP:______ Microalbuminuria:_____ Proteinuria:_______ |

1. **Bacteriological profile**

| Gram-positive | Gram-negative |  |
| --- | --- | --- |
| *Staphylococcus sp.*(1),  *S.aureus*(1a),  *S. epidermidis*(1b).  *Streptococcus sp.*(2)  *Enterococcus sp.*(3),  *E. faecalis*(3a),  *E.faecium*(3b) | *Enterobacteriaceae*(4)  *Escherichia sp.*(4a), *E. coli* (4a1), *E. hermanii* (4a2)  *Proteus sp.*(4b), *P. vulgaris* (4b1), *P. penneri* (4b2),  ***P. mirabilis***(4b3)  *Morganella sp.*(4c), *M. morganii* (4c1)  *Serratia sp.*(4d), *S. marcescens* (4d1)  *Enterobacter sp.*(4e), *E. cloacae* (4e1), *E. aerogenes* (4e2)  *Klebsiella sp.*(4f), K. pneumoniae(4f1), *K. oxytoca* (4f2)  *Citrobacter sp.*(4g), C*. freundii* (4g1), *C. kaseri* (4g2),  *providence sp*(4h), *P.rettgeri*(4h1), *P.stuarti*(4h2)  *Hafnia sp.*(4i), *Hafnia alvia*(4i1) | *Pseudomonas sp.*(5),  ***P. aeruginosa*(5a)**  *Acinetobacter sp.*(6), *A. baumannii* (6a)  **Other:**  **_______________** |

| Number of bacteria | 0, 1, 2 ,3, 4 |
| --- | --- |
| Bacteria 1 | Code:_______________________________ Gram: (0) Neg (1) Pos |
| Bacteria 2 | Code:_______________________________ Gram: (0) Neg (1) Pos |
| Bacteria 3 | Code:_______________________________ Gram: (0) Neg (1) Pos |
| Bacteria 4 | Code:_______________________________ Gram: (0) Neg (1) Pos |

| Multidrug-resistance | | | | |
| --- | --- | --- | --- | --- |
| *S.aureus*  Appendix 2  Yes No | *Enterococcus sp.*  Annex 3  Yes No | *Enterobacteriaceae*  Annex4  Yes No | *P. aeruginosa*  Annex 5  Yes No | *Acinetobacter sp.*  Annex 6  Yes No |

1. **Outcomes**

| Outcomes | Major amputation a. No b. Yes Death: a. No b. Yeas  Free amputation greater than 6 months from 1st contact. to. No b. Yes |
| --- | --- |
| Treatment time | Date of first contact:__________ Date of hospital admission: _________  Date of major amputation:__________ Hospital discharge date:____________  Date of last consultation:__________ |
